# Human diseases associated with defects in assembly of OXPHOS complexes

**DOI:** 10.1042/EBC20170099

**Published:** 2018-07-20

**Authors:** Daniele Ghezzi, Massimo Zeviani

**Affiliations:** 1 Molecular Neurogenetics, Foundation IRCCS Neurological Institute Besta, Milan, Italy; 2 Department of Pathophysiology and Transplantation, University of Milan, Milan, Italy; 3 Medical Research Council – Mitochondrial Biology Unit, University of Cambridge, Cambridge, U.K.

**Keywords:** Assembly Factor, mitochondrial disorders, mitochondrial respiratory chain, mitochondrial biogenesis, miochondrial complexes, oxidative phosphorylation

## Abstract

The structural biogenesis and functional proficiency of the multiheteromeric complexes forming the mitochondrial oxidative phosphorylation system (OXPHOS) require the concerted action of a number of chaperones and other assembly factors, most of which are specific for each complex. Mutations in a large number of these assembly factors are responsible for mitochondrial disorders, in most cases of infantile onset, typically characterized by biochemical defects of single specific complexes. In fact, pathogenic mutations in complex-specific assembly factors outnumber, in many cases, the repertoire of mutations found in structural subunits of specific complexes. The identification of patients with specific defects in assembly factors has provided an important contribution to the nosological characterization of mitochondrial disorders, and has also been a crucial means to identify a huge number of these proteins in humans, which play an essential role in mitochondrial bioenergetics. The wide use of next generation sequencing (NGS) has led to and will allow the identifcation of additional components of the assembly machinery of individual complexes, mutations of which are responsible for human disorders. The functional studies on patients’ specimens, together with the creation and characterization of *in vivo* models, are fundamental to better understand the mechanisms of each of them. A new chapter in this field will be, in the near future, the discovery of mechanisms and actions underlying the formation of supercomplexes, molecular structures formed by the physical, and possibly functional, interaction of some of the individual respiratory complexes, particularly complex I (CI), III (CIII), and IV (CIV).

## Introduction

The oxidative phosphorylation system (OXPHOS) consists of five multiheteromeric complexes embedded in the inner mitochondrial membrane. The first four complexes (complex I, CI; complex II, CII; complex III, CIII; complex IV, CIV or cytochrome *c* (cyt *c*) oxidase, COX), together with two mobile electron shuttles, ubiquinone (coenzyme Q, CoQ) and cyt *c*, form the respiratory chain (RC). Electron transport through RC generates energy, which is partly used by CI, CIII, and CIV to pump protons across the inner mitochondrial membrane thus creating an electrochemical potential (ΔP). ΔP constitutes the driving proton motive force for the production of ATP, operated by complex V (CV or ATP synthase), but also for heat production, Ca^++^ import inside mitochondria and homeostasis, protein translocation across mitochondrial membranes etc.

The genetic basis of the OXPHOS is unique, with the involvement of both nuclear and mtDNA. With the exception of CII, all the OXPHOS complexes contain subunits encoded by mtDNA: seven (MTND1, 2, 3, 4, 4L, 5, 6) are components of CI, one (cytochrome *b*) of CIII, three (MTCOI, II, III) of CIV, two (ATPase 6 and 8) of CV.

As a consequence, the assembly of each OXPHOS complex requires the insertion of mtDNA-encoded subunits into the inner membrane of mitochondria, in concert with tens of subunits encoded by nuclear genes; the synthesis and incorporation of several prosthetic groups that form the catalytic cores for redox reactions and the final formation of functionally active holocomplexes. Individual holocomplexes can also interact with each other forming mammoth structures called respiratory supercomplexes. A more detailed description of the role of these genes and the basic mechanisms of CI–V assembly is reported in a dedicated paper by Signes and Fernandez-Vizarra [1] in this issue.

Mitochondrial disorders are genetic defects affecting OXPHOS either ‘directly’ (e.g. OXPHOS subunits or assembly factors) or by impairing processes related to the proper formation of OXPHOS (e.g. mtDNA replication, transcription and translation, biosynthesis of RC cofactors, mitochondrial biogenesis,…). The former group is usually characterized by isolated biochemical defects, affecting a single complex, whereas the latter is typically associated with multiple OXPHOS deficiency. The structural and functional complexity of the biochemical pathways underpinning OXPHOS, explain the extreme heterogeneity of inherited mitochondrial disorders, which include a vast range of symptoms, severity, age of onset, progression, and outcome [2,3]. The prevalence of genetic OXPHOS defects is approximately 1:5000 live births, just considering mtDNA mutations [4], and even higher by including some frequent nuclear gene mutations [5,6]. Because OXPHOS is necessary for energy supply to virtually any cell, any organ can be affected by mitochondrial disease. However, the most common clinical presentations include the involvement of muscle, heart and brain, i.e. post-mitotic, specialized tissues, with high metabolic requests [7].

This review will be focussed on factors involved in assembly of human OXPHOS complexes, and associated with human diseases (Table 1). Any protein that plays a role in formation or stability of an OXPHOS complex, not being stable part of it, can be considered an ‘assembly factor’. However, in only a few cases the detailed mechanism of action of these factors has been elucidated, so that the definition of ‘assembly factor’ remains largely observational, based on the association between an assembly defect of a given complex with mutations in a particular gene product.

**Table 1:** Assembly factors of the OXPHOS with their (predicted) functions and related mitochondrial disease

Gene/protein	OMIM	(Predicted) function(s)*	Associated phenotypes
**CI assembly factors**			
NDUFAF1	606934	CI chaperone; transient interaction with early arm membrane intermediates (ND2 module)	Cardiomyoencephalopathy, lactic acidosis; leukodystrophy, neuropathy
NDUFAF2	609653	Stabilizer of late intermediate (N module)	Leukoencephalopathy with vanishing white matter, Leigh syndrome
NDUFAF3	612911	Interacts with some CI subunits and with NDUFAF4 (Q module)	Variable phenotypes: macrocephaly, severe muscle weakness, myoclonic seizures, brain leukomalacia; Leigh syndrome
NDUFAF4	611776	Interacts with some CI subunits and with NDUFAF3 (Q module)	Encephalopathy, antenatal cardiomyopathy, Leigh syndrome
NDUFAF5	612360	Probable methyltransferase of NDUFS7; early arm membrane assembly	Leigh syndrome, progressive spasticity
NDUFAF6	612392	Probable role in the assembly/stability of the Q module	Leigh syndrome; Acadian variant of Fanconi syndrome
NDUFAF7	615898	Methyltransferase of NDUFS2; stabilizer of early intermediate(s)	Pathologic myopia
ACAD9	611103	CI ND2 module assembler by the interaction with NDUFAF1, ECSIT and TMEM126B (MCIA)	Cardiomyopathy, encephalopathy, lactic acidosis, exercise intolerance
FOXRED1	613622	Mid-late stages of CI assembly (ND4 module)	Leigh syndrome; microcephaly and cardiomyopathy
TIMMDC1	615534	Assembly of membrane-embedded (ND1 module) and soluble arms of CI	Variable neurological phenotypes: Leigh syndrome; seizures, hypotonia, deafness, peripheral neuropathy, nystagmus
TMEM126B	615533	Assembly of the mature CI from the ND2 module 315- and 370-kDa subcomplexes	Exercise intolerance; cardiomyopathy and renal tubular acidosis
**CII assembly factors**			
SDHAF1	612848	Fe/S clusters insertion into SDHB	Leukoencephalopathy
SDHAF2	613019	Flavination of SDHA	Hereditary paraganglioma
**CIII assembly factors**			
BCS1L	603647	Incorporation of UQCRFS1	GRACILE syndrome, Bjornstad syndrome, encephalopathy, proximal tubulopathy and liver failure
TTC19	613814	Binding to fully assembled CIII dimer, role on UQCRFS1 turnover	Progressive encephalopathy, ataxia, psychiatric symptoms
LYRM7	615831	Binding and stabilization of UQCRFS1 and interaction with components of an Fe–S transfer complex for CIII	Leukcoencephalopathy, liver failure
UQCC2	614461	Interacts with UQCC1; synthesis of cyt *b* and the first steps of CIII assembly	Lactic acidosis, dysmorphic features; respiratory distress and seizures
UQCC3	616097	Cardiolipin-binding protein; stabilizer of CIII and CIII supercomplexes	Lactic acidosis, hypoglycemia, hypotonia, and delayed development
**CIV assembly factors**			
SURF1	185620	Formation of the early MTCO1 subcomplexes	Leigh syndrome
COA3/MITRAC12	614775	Interaction with early COX intermediates and assembly factors	Exercise intolerance and neuropathy
COA5/C2ORF64	613920	Involved in a very early step of the COX assembly	Fatal neonatal cardiomyopathy
COA7	615623	Unknown	Ataxia and neuropathy
COX14/c12orf62	614478	Coupling synthesis of MTCO1 with assembly into COX holoenzyme	Respiratory and neurologic distress, metabolic acidosis and neonatal death
COX20/FAM36A	614698	Involved in early steps of the COX assembly; interaction with MTCO2	Ataxia and muscle hypotonia, dystonia-ataxia
PET100	614770	Involved in intermediate stage of COX assembly	Psychomotor delay, seizures, hypotonia, and Leigh syndrome
PET117	614771	Coupling Heme *a* synthase activity to COX assembly. Interaction with PET100	Neurodevelopmental regression
APOPT1	616003	Unknown	Leukoencephalopathy
**Copper incorporation**			
COA6	614772	Copper homeostasis and transport to CIV	Fatal infantile cardioencephalomyopathy
SCO1	603644	Incorporation of copper atoms in the catalytic sites of the nascent CIV	Infantile encephalopathy, neonatal hepatopathy, ketoacidotic comas
SCO2	604272	Incorporation of copper atoms in the catalytic sites of the nascent CIV	Infantile cardioencephalomyopathy, myopia, CMT
**Heme biosynthesis**			
COX10	602125	Heme A synthesis (conversion of heme *b* into heme *o*)	Leigh syndrome, proximal renal tubulopathy, hypertrophic cardiomyopathy, sensorineural deafness, metabolic acidosis
COX15	603646	Heme A synthesis (conversion of heme *o* into heme *a*)	Infantile cardiomyopathy, Leigh syndrome
**CV assembly factors**			
ATPAF2	608918	F1 chaperone; essential for assembly of α + β heterooligomer	Degenerative encephalopathy, connatal lactic acidosis, methyl glutaconic aciduria
TMEM70	612418	Assembly of F1; structure of cristae	Neonatal encephalocardiomyopathy
**Fe–S biosynthesis**			
BOLA3	613183	Specific Fe–S cluster targetting factor	Epileptic encephalopathy, cardiomyopathy, spasticity (MMDS2)
FDXR	103270	Ferredoxin reductase	Auditory neuropathy, optic atrophy
FXN	606829	Iron chaperone	Friedreich’s ataxia
GLRX5	609588	Fe–S cluster transfer to apoproteins	Sideroblastic anemia, spasticity
IBA57	615316	Required for [4Fe–4S] cluster assembly	Leukodystrophy, hypotonia, dysmorphism, SPOAN (MMDS3)
ISCA1	611006	Required for [4Fe–4S] cluster assembly	Leukodystrophy, epilepsy (MMDS5)
ISCA2	615317	Required for [4Fe–4S] cluster assembly	Leukodystrophy (MMDS4)
ISCU	611911	Scaffold protein for Fe-S cluster synthesis	Myopathy, hypertrophic cardiomyopathy
LYRM4/ISD11	613311	Fe-S protein biogenesis desulphurase interacting protein	Respiratory distress, hypotonia, hepatopathy
NFS1	603485	Cysteine desulphurase	Lactic acidosis, hypotonia, multisystem organ failure
NFU1	608100	Scaffold protein for [4Fe–4S] cluster synthesis	Hypotonia, leukodystrophy, epilepsy (MMDS1)
NUBPL	613621	Facilitates the assembly of Fe–S cofactors and subunits in CI	Leukodystrophy, myopathy, ataxia (CI deficiency)
**Cofactor and cytochrome biosynthesis**			
HCCS	300056	Synthesis of cyt *c1* and cyt *c*	MIDAS
CYCS	123970	cyt *c*	Thrombocytopenia
FLAD1	610595	Synthesis of FAD	Lipid storage myopathy
**CoQ10**			
ADCK3/COQ8A	606980	CoQ10 biosynthesis	Cerebellar ataxia
ADCK4/COQ8B	615567	CoQ10 biosynthesis	Nephrotic syndrome, proteinuria
COQ2	609825	Parahydroxybenzoate-polyprenyltransferase	Encephalomyopathy; cardiomyopathy and renal failure; ataxia; Leigh syndrome; isolated myopathy
COQ4	612898	CoQ10 biosynthesis	Cardiac or neurologic involvement
COQ6	614647	Flavin-dependent monooxygenase	Nephrotic syndrome, seizures
COQ7	601683	Di-iron oxidase	Neonatal complex multisystem disorder
COQ9	612837	CoQ10 biosynthesis	Encephalopathy, microcephaly
PDSS1	607429	Trans-prenyltransferase (subunit 1)	Early-onset multisystem disorder
PDSS2	610564	Trans-prenyltransferase (subunit 2)	Fatal encephalomyopathy and nephrotic syndrome

Abbreviations: CMT, Charcot–Marie–Tooth; Fe–S, iron–sulphur; FOXRED, FAD-dependent oxidoreductase-containing domain 1; GRACILE, growth retardation, aminoaciduria, cholestasis, iron overload, lactic acidosis, early death; MIDAS, microphthalmia, dermal aplasia and sclerocornea; MMDS, multiple mitochondrial dysfunctions syndrome; NUBPL, nucleotide-binding protein like; SCO, synthesis of cytochrome oxidase 1; SCO2, synthesis of cytochrome oxidase 2; SDHAF, SDH assembly factor 1; SPOAN, spastic paraparesis, peripheral neuropathy  ±  optic nerve atrophy; UQCRFS1, Rieske Fe–S protein.

* A detailed description of the functions of the assembly factors is reported in a dedicated paper by Signes and Fernandez-Vizarra [1] in this issue.

Several genes encoding enzymes or proteins with a role in synthesis of prosthetic groups and cofactors have been classified as ‘assembly factors’ in the past: e.g. *COX10* and *COX15*, encoding enzymes involved in the terminal steps of the biosynthesis of hemes a and a3; synthesis of cytochrome oxidase 1 and 2 (*SCO1* and *SCO2*), involved in cellular copper homeostasis. Clinical presentations associated with mutations in these genes are briefly described in this manuscript. Several enzymes, chaperones and transporters are necessary for the biosynthesis of the iron–sulphur (Fe–S) clusters and the corresponding genetic defects are usually associated with multiple biochemical defects involving RC complexes containing Fe–S centers, namely cI, cII and cIII. Recent reviews describe in detail this group of diseases [8,9]. Moreover, proteins/enzymes related to the synthesis of the RC electron shuttles, CoQ and cyt *c*, have been sometimes considered as ancillary factors for the OXPHOS system; the human diseases associated with CoQ deficiency have been reviewed elsewhere [10]. Examples of these genes are reported in Table 1 but will not be described in detail in this review.

## Human diseases associated with CI deficiency (MIM 252010)

Approximately one-third of all cases with mitochondrial disorders are biochemically characterized by an isolated CI deficiency [11,12]. A large percentage still lacks a molecular diagnosis, because of the complexity of this huge enzyme, its dual genetic origin, and the incomplete information about its assembly, turnover, and regulation. The clinical presentations are highly heterogeneous, including, for children, Leigh syndrome (LS), neonatal cardiomyopathy with lactic acidosis, fatal infantile lactic acidosis (FILA), macrocystic leukoencephalopathy, or isolated myopathy [13,14]. Similar to other CI defective conditions, mutations in CI assembly factors cause a wide range of clinical disorders.

### NDUFAF1 (MIM 606934)

NDUFAF1 (previously known as CIA30) has been shown to interact with mitochondrial and nuclear CI subunits [15] and is physically associated with two assembly intermediates [16]. Mutations in *NDUFAF1* were reported in two unrelated patients with cardiomyoencephalopathy, lactic acidosis, and reduced levels of CI [15,17]. Both patients developed hypertrophic cardiomyopathy in infancy after a viral illness. More recently, *NDUFAF1* mutations were found in a child with leukodystrophy, peripheral neuropathy, and CI deficiency [18].

### NDUFAF2 (MIM 609653)

A stop mutation of *NDUFAF2* (*B17*.2L or *NDUFA12L*) was detected in a patient with progressive leukoencephalopathy with vanishing white matter, and impaired CI assembly [19]. A different mutation, which affects the first methionine, was found in two infants with hypotonia, nystagmus, and ataxia [20] associated with reduced CI activity in muscle. Additional homozygous *NDUFAF2* mutations were identified in LS patients [21,22].

### NDUFAF3 (MIM 612911)

Mutations in *NDUFAF3/C3ORF60* were found in three families with CI deficiency associated with a spectrum of severe phenotypes: a fulminant syndrome dominated by muscle hypertonia in the first, macrocephaly and severe muscle weakness in the second, myoclonic epilepsy and leukomalacia in the third. All patients died before 6 months of age [23]. LS has been recently described as a clinical feature of *NDUFAF3* deficiency [24].

### NDUFAF4 (MIM 611776)

A homozygous mutation in *NDUFAF4/C6ORF66* was associated with severe CI deficiency in five consanguineous patients presenting with infantile encephalopathy and in one unrelated case of antenatal cardiomyopathy. Reduction in fully assembled CI and accumulation of assembly intermediates was observed in patients’ mitochondria [25].

### NDUFAF5/C20ORF7 (MIM 612360)

A homozygous mutation in an anonymous gene, *C20ORF7* (now *NDUFAF5*), was identified in a lethal neonatal form of CI deficiency by homozygosity mapping followed by candidate gene analysis [26]. Additional NDUFAF5 mutations were later found in subjects with LS [27,28]. Interestingly, some patients show combined deficiency of CI and CIV, suggesting for NDUFAF5 an additional role in CIV assembly or in the formation of CI–CIV supercomplexes.

### NDUFAF6 (MIM 612392)

A homozygous missense mutation in a conserved residue of NDUFAF6 was associated with LS with isolated CI deficiency [29]. Later, biallelic missense mutations in *NDUFAF6* were identified in children with LS due to mitochondrial complex I deficiency [30–32]. Notably, a homozygous ultra-rare non-coding variant (rs575462405) located in intron 2 of *NDUFAF6* was found in nine patients with the Acadian variant of Fanconi syndrome. This variant impairs *NDUFAF6* splicing and affected kidney and lung showed specific loss of the mitochondria-located *NDUFAF6* isoforms [33].

### NDUFAF7 (MIM 615898)

A heterozygous mutation in *NDUFAF7* was recently proposed as causative in a Chinese family with pathologic myopia. This variant segregated within the family; impaired complex I activity and decreased ATP levels were found in cultured patient’s cells [34].

### ACAD9 (MIM 611103)

Mutations in *ACAD9* are quite frequent and associated with infantile hypertrophic cardiomyopathy, encephalopathy, and lactic acidosis [35–37]. All patients had a reduction in CI enzymatic activity and assembly. Severe neonatal presentations [38] and multiorgan involvement, with liver and kidney damage [39], broaden the phenotypic spectrum of *ACAD9* disease. Most of the ACAD9 mutant cells and patients respond to riboflavin treatment, with partial correction of CI deficiency and clinical improvement [35,40], possibly because ACAD9 is an FADH_2_-dependent acyl-CoA dehydrogenase. Nevertheless, non-responsive patients have been reported [41]. The surviving patients often develop delayed-onset neurologic or muscular symptoms [37]. Patients with missense mutations are usually mildly affected, with childhood onset cardiomyopathy [42] or lifetime exercise intolerance and lactic acidosis [40,43].

ACAD9 displays a β-oxidative activity *in vitro* but fatty acid β-oxidation has been reported as normal in most patients with *ACAD9* mutations. However, the enzymatic activity of ACAD9, required for full fatty acid oxidation capacity, was suggested to be important in cells expressing high levels of ACAD9 (neurones and liver), thus impairment of this function may contribute to the phenotype [44].

### FOXRED1 (MIM 613622)

FAD-dependent oxidoreductase-containing domain 1 (*FOXRED1*) was identified by gene screening of CI-defective patients with LS [21] or encephalocardiomyopathy [45]. A homozygous missense mutation was identified in a subject with epilepsy and severe psychomotor retardation, associated with severe reduction in CI and a mild decrease in CII. The authors suggested that FOXRED1 may play a role in the assembly of two flavoprotein-containing OXPHOS complexes [46].

### TIMMDC1 (MIM 615534)

A homozygous intronic *TIMMDC1* mutation was identified in three unrelated patients with mitochondrial CI deficiency [47]; the nucleotide change results in aberrant splicing and premature termination. Both TIMMDC1 RNA and protein showed severely decreased expression. All patients had severe early-onset neurologic dysfunctions (e.g. hypotonia, failure to thrive, sensorineural deafness, peripheral neuropathy, nystagmus, seizures).

### TMEM126B (MIM615533)

Biallelic mutations in *TMEM126B* were reported in patients with CI deficiency and exercise intolerance affecting only skeletal muscle [48] and in one subject presenting a more severe phenotype with hypertrophic cardiomyopathy and renal tubular acidosis [49].

### NUBPL/Ind1 (MIM 613621)

Fe–S clusters are present in CI, CII and CIII, and several enzymes are required for their biosynthesis (Table 1). However NUBPL (nucleotide-binding protein like) has a specific role in the incorporation of Fe–S centers into CI [50]. Compound heterozygous *NUBPL* mutations were first identified in a single case, presenting with mitochondrial encephalopathy and CI deficiency [21] and then in six subjects with the same biochemical defect and a characteristic leukoencephalopathic pattern on brain MRI [51].

## Human diseases associated with CII deficiency (MIM 252011)

Isolated defect of CII is a rare biochemical finding, observed in <10% of OXPHOS defective cases [52,53]. Two main clinical presentations have been reported: mitochondrial encephalomyopathy and familial paragangliomas.

In the first group, LS is the most common clinical and neuropathological presentation; additional phenotypes include myopathy, encephalopathy, leukodystrophy, and isolated cardiomyopathy. The pathogenesis of CII-associated paragangliomas/pheochromocytomas remains to be explained. The most widely accepted hypothesis is based on induction of the hypoxia program that switches energy metabolism from mitochondrial respiration to glycolysis [54].

Mutations in genes encoding for either structural subunits or assembly factors have been described (*SDHA, SDHB, SDHD*, and SDH assembly factor 1 (*SDHAF1*) for mitochondrial diseases; *SDHD, SDHC, SDHB, SDHA*, and *SDHAF2* for hereditary paragangliomas). Defects in several factors involved in FAD (e.g. *FLAD1*) [55] or Fe–S cluster synthesis (e.g. *IBA57, ISCU*) [56,57] can impair assembly and activity of CII, as well as of other Fe–S or FAD-dependent enzymes; however, only four are presently known as specific CII assembly factors (SDHAF1–4). Mutations in two of them, namely SDHAF1 [53] and SDHAF2 [58], have been associated with human pathologies.

### SDHAF1 (MIM612848)

SDHAF1, standing for SDH Assembly Factor 1, is a small protein containing an LYR motif characteristic of proteins involved in Fe–S metabolism [53]. SDHAF1 was shown to contribute to Fe–S cluster incorporation into the CII subunit SDHB [59]. Mutations in this protein are associated with drastic decrease in CII activity and content in both humans and yeast. Homozygous missense (and one nonsense) mutations in *SDHAF1* have been identified in affected subjects from six families, presenting with leukoencephalopathy; a peculiar hallmark was accumulation of lactate and succinate in the white matter [53,59,60]. To date, no mutation in *SDHAF1* has been reported in patients with paraganglioma [61].

### SDHAF2 (MIM 613019)

The function of SDHAF2 is likely related to the flavination of the subunit SDHA [58]. The binding of FAD to SDHA is probably a self-catalytic process, but requires that the imported SDHA subunit is properly refolded, forming the FAD-binding pouch. Sdhaf2/SDHAF2 could be a chaperone responsible for this step [62].

A germline missense mutation in *SDHAF2*, G78R, has been reported in two large families with hereditary, multiple head and neck paragangliomas (PGL2). Haplotype analysis indicated that the G78R occurred independently in the two families [63]. The G78 residue is highly conserved and the mutant R78 was demonstrated to alter its interaction with the SDHA subunit [58]. Additional patients harboring nonsense or heterozygous *SDHAF2* mutations, presented with benign head and neck PGLs [61,64]. A variant in 3′-UTR was reported in two unrelated subjects with adrenal pheochromocytoma [65].

## Human diseases associated with CIII deficiency (MIM 124000)

CIII defects are rare, compared with those of CI or CIV. CIII deficiency is caused by recessively inherited mutations affecting nuclear encoded structural subunits or assembly factors, and is associated with a wide range of clinical presentations and reduced CIII activity/amount [66]. CIII deficiency may also, and relatively frequently, be due to mutations in the mtDNA gene *MTCYB*, typically associated with myopathy and exercise intolerance.

In the recent years, the introduction of next generation sequencing (NGS) techniques, together with the discovery of additional assembly factors in yeast, has led to the identification of more disease genes encoding CIII-assembly factors, in addition to mutations of BCS1L, which were discovered in 2001 [67].

### BCS1L (MIM 603647)

Several *BCS1L* gene mutations have been reported in CIII deficiency, associated with different clinical presentations ranging from multisystem involvement including neonatal proximal tubulopathy, hepatopathy, and encephalopathy, to isolated neurological syndrome with long-term survival [67–69]. Specific syndromes can be caused by *BCS1L* mutations. The acronym GRACILE stands for growth retardation, aminoaciduria, cholestasis, iron overload, lactic acidosis and early death, and designates an infantile condition caused by a specific BCS1L mutation, S78G, which is part of the Finnish disease heritage [70]. A less-severe phenotype associated with *BCS1L* missense mutations is Björnstad syndrome, characterized by neurosensory hearing loss and abnormally curly and brittle hair (*pili torti*). The clinical heterogeneity could be linked to the functional domain affected by the different missense mutations [71]. Few nonsense mutations as well as variants in splice sites and in the 5’-UTR of the *BCS1L* mRNA have also been found [72,73]. All *BCS1L* mutations are associated with isolated CIII deficiency (rarely in combination with reduced CIV and CI activities) and reduced amount of Rieske Fe–S protein (UQCRFS1) incorporated into CIII.

### TTC19 (MIM 613814)

*TTC19* mutations have been reported in a few patients with heterogeneous phenotypes ranging from early onset neurodegenerative disorders [74,75] to adult forms with psychiatric manifestations and cerebellar ataxia [76,77]. In *TTC19*-mutant cases, ataxia and impairment of cortical functions leading to language or cognitive regression are the clinical hallmarks of infantile-onset forms, whereas psychiatric symptoms are typical of juvenile-adult forms. MRI patterns are consistent with Leigh or Leigh-like syndrome. Decreased cIII activity was present in almost all patients reported to date, while lactic acidosis seems not to be a reliable biomarker [78]. Notably, most of the *TTC19* mutations are nonsense or frameshift changes; a few missense mutations have been described, associated with the absence or strong reduction in the protein [79].

### LYRM7 (MIM 615831)

As SDHAF1, LYRM7 contains an LYR motif, the molecular signature of proteins involved in the delivery of Fe–S clusters [80]. A homozygous missense mutation was found in a CIII-deficient patient who showed severe, acute, and ultimately fatal neurologic decompensation and regression after having had 20-month long normal development [81]. Six different homozygous mutations were later reported in patients with defects of mitochondrial complex III and a similar and distinct pattern of leukoencephalopathy on brain imaging [82]. A homozygous, truncating, mutation in *LYRM7* was found in a child with complex III defect and acute liver dysfunction with lactic acidosis [83], a phenotype resembling *BCS1L* patients.

### QCC2 (MIM 614461)

A homozygous splice site mutation in *UQCC2* was first described in a boy with lactic acidosis, mild dysmorphic features, delayed neurological development and sensorineural hearing impairment. This subject had CIII deficiency but also presented secondary reduction in CI and CIV activities [84]. Recently, a second case was published: a girl with respiratory distress and severe epileptic seizures, born after a pregnancy complicated by intrauterine growth retardation and oligohydramnios, who died at 1 month of age. Two homozygous missense variants in *UQCC2* were identified, and a severe reduction in UQCC2 protein was demonstrated [85].

### UQCC3 (MIM 616097)

A homozygous missense mutation in *UQCC3* was identified in a patient diagnosed with isolated CIII deficiency, displaying lactic acidosis, hypoglycemia, hypotonia, and delayed development without dysmorphic features [86]. UQCC3 was shown to be a cardiolipin-binding protein involved in the stabilization of CIII-containing supercomplexes [87].

CIII defects with different genetic bases, with the exception of *TTC19* deficiency, often present a combined RC deficiency. Besides CIII, CI and, in some cases, CIV activities are decreased [84,88]. The presence of fully assembled CIII is probably necessary for the stability or assembly of CI and CIV, which might be related to respirasome/supercomplex formation.

## Human diseases associated with CIV deficiency (MIM 220110)

Together with defects of CI, CIV (or COX) deficiencies are quite common biochemical hallmarks of mitochondrial disease. In infancy, the most frequent manifestation of isolated and severe COX deficiency is LS, but other encephalocardiomyopathy phenotypes are known. Several mutations of mtDNA tRNA genes are associated with maternally inherited COX defects. Conversely, only a few mutations in the genes encoding structural COX subunits (either mtDNA- or nuclear-encoded, e.g. MTCO1, MTCO2, MTCO3, COX6B, COX7B, COX8A) have been reported to date, suggesting that most of the mutations in structural components of CIV are incompatible with extrauterine life. Accordingly, the most common defects of COX are due to mutations in nuclear DNA genes coding for assembly factors or for enzymes/proteins with a role in biosynthesis/incorporation of CIV prostetic groups.

### SURF1 (MIM 185620)

Mitochondrial protein SURF1 is a specific assembly factor of COX, but its function is poorly understood. Mutations in *SURF1* are the most common cause of LS associated with COX deficiency [89]. This association is specific, and is partly explained by the observation that almost all the SURF1 mutations reported to date cause the complete absence of the protein. Very few missense mutations have been detected [90], sometimes in association with less severe phenotypes [91]. Nevertheless, no clear genotype–phenotype correlations are detectable amongst these patients [92]. Even amongst subjects who showed an unusual long survival, COX activity was not detectable or strongly reduced, including cases harboring a *SURF1* variant that abolish the initiation codon [93]. In addition to LS, a peculiar phenotype that has been associated with *SURF1* mutations is Charcot–Marie–Tooth disease type 4K, an autosomal recessive demyelinating peripheral neuropathy characterized by onset in the first decade of distal muscle weakness and atrophy, with muscle CIV deficiency [94].

In *SURF1* null human samples [89], fully assembled, functionally active CIV is found in residual amounts, suggesting partial functional redundancy. Studies based on mouse models revealed tissue-specific and species-specific differences in COX biogenesis and COX ability to incorporate into respiratory supercomplexes, supporting the view that COX assembly is much more dependent on SURF1 in humans than in mice [95].

### COA3/MITRAC12 (MIM 614775)

COA3 was identified in immunoprecipitation studies as a protein interacting with central CIV subunits, e.g. MTCO1, and assembly factors, e.g. SURF1 and COX14 [96]. Compound heterozygous mutations in *COA3* were identified in a woman with severe cIV deficiency in muscle but a relatively mild phenotype characterized by exercise intolerance, peripheral neuropathy, obesity, and short stature [97]. The authors suggested a tissue-specific defect mainly affecting muscle.

### COA5/C2ORF64 (MIM 613920)

COA5 or C2ORF64, is the ortholog of PET191, a yeast COX assembly factor. A homozygous mutation in *C2ORF64* was described in two siblings affected by fatal neonatal cardiomyopathy. The activity and amount of CIV was severely reduced in patient fibroblasts and heart muscle, with accumulation of a small assembly intermediate containing subunit MTCO1 but not MTCO2, COX4, or COX5a, indicating that C2ORF64 is involved in a very early step of COX assembly [98].

### COA7 (MIM 615623)

COA7 is a mitochondrial protein, putative COX assembly factor, without a yeast ortholog.

Biallelic pathogenic *COA7* mutations were identified in a young woman, affected by early onset, progressive severe ataxia and peripheral neuropathy, mild cognitive impairment and a cavitating leukodystrophy of the brain. Biochemical analysis revealed the presence of isolated CIV deficiency in skin fibroblasts and skeletal muscle [99].

### COX14/c12orf62 (MIM 614478)

By investigating three siblings with severe congenital lactic acidosis and dysmorphic features associated with a COX-assembly defect, a homozygous mutation in *C12ORF62* (now *COX14*) was found as the cause of the disease [100]. Further studies suggested that COX14 is required for co-ordination of the early steps of COX assembly with the synthesis of MTCO1 [100] and demonstrated an interaction between COX14 and MTCO1 [101].

### COX20/FAM36A (MIM 614698)

COX20 associates with MTCO2 and is required for its stability; moreover, it appears to act in the early steps of CIV assembly. A homozygous mutation in *COX20* was found by analyzing candidate genes in the mutational screening of a patient with growth retardation, hypotonia, and cerebellar ataxia [102]. The same mutation was identified in two siblings with dystonia-ataxia syndrome. They presented with a combination of childhood-onset cerebellar ataxia, dystonia, and sensory axonal neuropathy; biochemical analyses revealed CIV and CoQ10 deficiency in a muscle biopsy [103]. All these patients were of Turkish origin.

### PET100 (MIM 614770)

PET100 is a mitochondrial inner protein, initially described in yeast as required for the assembly of CIV [104]. A homozygous mutation affecting the initiation codon was identified in ten affected subjects of Lebanese descent, due to a founder effect. The patients presented with profound psychomotor delay since early infancy, seizures, hypotonia, and LS, associated with reduction in CIV activity and amount of the holoenzyme [105]. A nonsense *PET100* mutation caused fatal infantile lactic acidosis, again associated with isolated CIV deficiency [106].

### PET117 (MIM 614771)

PET117 is a small protein that has previously been predicted as a CIV assembly factor [101]. A homozygous nonsense mutation was detected in two sisters with a mitochondrial disease characterized by lesions in the medulla oblongata, and an isolated CIV deficiency with reduced levels of CIV subunits [107].

### APOPT1 (MIM 616003)

APOPT1 is a mitochondrial protein deemed to initiate apoptosis by triggering release of cyt *c* [108]; since its levels increase after oxidative challenge, a role in detoxification of reactive oxygen species has been proposed [109]. *APOPT1* mutations were identified in patients with brain MRI pattern characterized by cavitating leukodystrophy. The clinical features of the mutant subjects varied widely from acute neurometabolic decompensation to subtle neurological signs; all presented a chronic, long-surviving clinical course [109].

In addition to specific assembly factors, ancillary proteins are necessary for incorporation of hemes (a, a3) and copper atoms (CuA, CuB) into catalytic subunits of CIV. Mutations in the corresponding genes are associated with human diseases characterized by CIV deficiency.

### SCO1 (MIM603644) and SCO2 (MIM 604272)

*SCO1* and *SCO2* promote the insertion of Cu^++^ atoms in the catalytic sites CuB and CuA of MTCO1 and MTCO2 subunits. Mutations in *SCO2* were initially found in infants with fatal cardioencephalomyopathy and COX deficiency [110]. Heart hypertrophy in patients with *SCO2* mutations is usually severe, whereas brain involvement may vary, from LS-like to spinal muscular atrophy-like presentations [111]. Very recently, recessive *SCO2* mutations have been reported in subjects with axonal polyneuropathy (Charcot–Marie–Tooth disease type 4) [112]. A peculiar dominant phenotype was associated with a heterozygous nonsense mutation segregating with disease in a large four-generation family with high-grade myopia [113].

Mutations in *SCO1* are extremely rare and have been found in a single large family with multiple cases of neonatal hepatopathy, severe ketoacidosis, and COX deficiency [114]. Other *SCO1* cases showed fatal encephalopathy, with or without cardiomyopathy and hepatomegaly [115,116].

### COX10 (MIM 602125) and COX15 (MIM 603646)

COX10 and COX15 are enzymes involved in the terminal steps of the biosynthesis of hemes a and a3. Mutations in *COX10* are associated with a spectrum of conditions including LS, encephalopathy with proximal tubulopathy, cardiomyopathy, sensorineural deafness, and metabolic acidosis [117,118]. Mutations of *COX15* can cause fatal infantile hypertrophic cardiomyopathy [119] and rapidly progressive or protracted LS [120].

### COA6/C1orf31 (MIM 614772)

COA6 binds copper, interacts with SCO1 and can associate with MTCO2 [121]. Recessive mutations of *COA6* have been associated with fatal infantile cardioencephalomyopathy [122,123]

## Human diseases associated with CV deficiency

Mitochondrial CV or ATP synthase deficiency due to nuclear genes mutations is often characterized by neonatal-onset hypotonia and hypertrophic cardiomyopathy; lactic acidosis and 3-methylglutaconic aciduria are typical biochemical hallmarks of these diseases. Few disease-causing nuclear genes have been identified so far, encoding assembly factors (ATPAF2, TMEM70) or structural subunits (ATP5E, ATP5A1) [124]. Furthermore, maternally transmitted CV deficiency can be caused by mutations in the two mtDNA genes *MTATP6* or *MTATP8*. Heteroplasmic missense mutations in *MTATP6* [125,126] are associated with adult-onset NARP (neuropathy, ataxia, and retinitis pigmentosa) or maternally inherited LS (MILS). Additional rare phenotypes associated with *MTATP6* mutations have been reported, including mitochondrial myopathy, lactic acidosis, and sideroblastic anemia (MLASA) [127]; adult-onset spinocerebellar ataxia [128]; motor neurone syndrome [129]. A single patient with hypertrophic cardiomyopathy carried a nonsense mutation in MTATP8 [130], whereas few patients with hypertrophic cardiomyopathy and heart failure [131] or ataxia and peripheral neuropathy [132] harbored a heteroplasmic mtDNA variant, resulting in concurrent substitutions in the overlapping *MTATP6* and *MTATP8* genes.

### TMEM70 (MIM 612418)

Mutations in *TMEM70* are the most frequent cause of CV deficiency [133,134]. Mutations in *TMEM70* were originally found in patients, mostly of Roma origin, with neonatal encephalocardiomyopathy and isolated CV deficiency [135]. The prevalent homozygous mutation, an A-to-G transition in intron 2 of *TMEM70*, results in aberrant splicing and loss of the mRNA transcript; this common variant is however associated with highly variable clinical severity, possibly due to individual variations in nonsense-mediated RNA decay systems. Several additional patients with various ethnic backgrounds and different mutations have been reported. The most frequent symptoms at onset are respiratory distress, hypotonia, cardiomyopathy, poor feeding, and psychomotor delay [136,137], often associated with short stature, microcephaly, and facial dysmorphism. Typical biochemical findings are lactic acidosis, 3-methylglutaconic aciduria, and hyperammonaemia. The outcome of this multisystem disease depends mainly on adequate management of neonatal hyperammonemic crises.

Samples from patients with mutations in *TMEM70* showed small amounts of CV holocomplex and the presence of traces of free F1 catalytic particle of the complex [138]. Ultrastructural studies in *TMEM70*-mutant samples showed swollen degenerated mitochondria, cristae aggregation, and formation of concentric membrane rings [136,139]. Moreover, not only CV deficiencies but also impairment of other OXPHOS complexes have been described in *TMEM70*-mutant subjects. These findings indicated that CV impairment could indirectly alter other RC complex activities by disrupting the mitochondrial cristae structure, for instance affecting the integrity of mitochondrial nucleoids and hence mtDNA replication and expression.

### ATPAF2 (MIM 608918)

ATPAF1 and ATPAF2 are chaperones interacting with subunits β and α of the peripheral F1 catalytic particle, essential for assembly of the α+β heterooligomer [140,141]. To date, only one case of CV deficiency has been referred to a homozygous missense *ATPAF2* mutation associated with degenerative encephalopathy, connatal lactic acidosis, and methyl-glutaconic aciduria [142]. The amount of fully assembled CV was low, but no subassembly intermediates were detected, suggesting that ATPAF2 acts very early during CV assembly [138].

## Mitochondrial supercomplexes

The vision of the OXPHOS complexes as isolated enzymes in the IM has been replaced by a model in which they associate with each other to form supramolecular structures, called supercomplexes. Supercomplexes have been shown to be functionally active *in vitro*, and this has led to the hypothesis that they could facilitate substrate channeling and electron transfer, and required for forming stable OXPHOS complexes [143–145]. Proteins requested for supercomplex assembly may exclusively include assembly factors that help assemble supercomplexes after the assembly of individual complexes has taken place or assembly factors shared between different OXPHOS complexes. Indeed, multiple OXPHOS deficiency or impairment of supercomplexes have been already reported in some cases harboring mutations in genes encoding known assembly factors for ‘single’ complexes: e.g. *NDUFAF2* [19], *NDUFAF5* [28], *UQCC2* [85], *UQCC3* [87], *COA7* [99].

In addition to their recognized biological role, it is expected that in the near future there will be increasing evidence about the significance of mitochondrial supercomplexes, and their as yet unknown assembly factors, also in medical contexts.

## Final remarks

Multiheteromeric complexes like the OXPHOS complexes need to be assembled through a finely tuned process requiring many dedicated chaperones or assembly factors. The fact that four out of five OXPHOS complexes contain subunits encoded by two different genomes (the nuclear and mtDNA) further complicates the process. Thus, it is not surprising that impairment in OXPHOS complex assembly is linked to human diseases. Defects of genes encoding several assembly factors for all OXPHOS complexes are responsible for a wide variety of pathological conditions, mainly affecting tissues/organs with high energetic demand as for other mitochondrial disorders. At biochemical analysis, these genetic diseases are typically associated with isolated deficiencies in single specific OXPHOS complexes.

Thanks to the wide use of NGS in the diagnostic workflow of patients with clinical and/or biochemical features suggestive for a mitochondrial disorder, the list of human diseases associated with defects in assembly of OXPHOS complexes will probably grow up with the identification of mutations in known assembly factors still without an associated pathological phenotype or in newly discovered assembly factors.

## Summary

- Assembly factors of the mitochondrial oxidative phosphorylation (OXPHOS) system that have been reported in the literature as responsible for many mitochondrial diseases in humans.
- Importantly, the investigation of patients with these genetic defects has allowed the identification of several new assembly factors and contributed quite substantially to the elucidation of the molecular mechanism in some of them.

## Abbreviations

COX: cytochrome *c* oxidase
CoQ: coenzyme Q
cyt *c*: cytochrome *c*
CI: complex I
CII: complex II
CIII: complex III
CIV: complex IV
CV: complex V
Fe–S: iron–sulphur
FOXRED: FAD-dependent oxidoreductase-containing domain 1
LS: Leigh syndrome
NGS: next generation sequencing
NUBPL: nucleotide-binding protein like
OXPHOS: oxidative phosphorylation system
RC: respiratory chain
SCO1: synthesis of cytochrome oxidase
SDHAF1: SDH assembly factor 1
ΔP: electrochemical potential
